# New Insights into the Methodology of L-Arginine-Induced Acute Pancreatitis

**DOI:** 10.1371/journal.pone.0117588

**Published:** 2015-02-17

**Authors:** Balázs Kui, Zsolt Balla, Béla Vasas, Eszter T. Végh, Petra Pallagi, Eszter S. Kormányos, Viktória Venglovecz, Béla Iványi, Tamás Takács, Péter Hegyi, Zoltán Rakonczay

**Affiliations:** 1 First Department of Medicine, University of Szeged, Szeged, Hungary; 2 Department of Pathology, University of Szeged, Szeged, Hungary; 3 Department of Pharmacology and Pharmacotherapy, University of Szeged, Szeged, Hungary; 4 Hungarian Academy of Sciences-University of Szeged, Translational Gastroenterology Research Group, Szeged, Hungary; University of Valencia, SPAIN

## Abstract

Animal models are ideal to study the pathomechanism and therapy of acute pancreatitis (AP). The use of L-arginine-induced AP model is nowadays becoming increasingly popular in mice. However, carefully looking through the literature, marked differences in disease severity could be observed. In fact, while setting up the L-arginine (2×4 g/kg i.p.)-induced AP model in BALB/c mice, we found a relatively low rate (around 15%) of pancreatic necrosis, whereas others have detected much higher rates (up to 55%). We suspected that this may be due to differences between mouse strains. We administered various concentrations (5–30%, pH = 7.4) and doses (2×4, 3×3, or 4×2.5 g/kg) of L-arginine-HCl in BALB/c, FVB/n and C57BL/6 mice. The potential gender-specific effect of L-arginine was investigated in C57BL/6 mice. The fate of mice in response to the i.p. injections of L arginine followed one of three courses. Some mice (1) developed severe AP or (2) remained AP-free by 72 h, whereas others (3) had to be euthanized (to avoid their death, which was caused by the high dose of L-arginine and not AP) within 12 h., In FVB/n and C57BL/6 mice, the pancreatic necrosis rate (about 50%) was significantly higher than that observed in BALB/c mice using 2×4 g/kg 10% L–arginine, but euthanasia was necessary in a large proportion of animals, The i.p. injection of lower L-arginine concentrations (e.g. 5–8%) in case of the 2×4 g/kg dose, or other L-arginine doses (3×3 or 4×2.5 g/kg, 10%) were better for inducing AP. We could not detect any significant differences between the AP severity of male and female mice. Taken together, when setting up the L-arginine-induced AP model, there are several important factors that are worth consideration such as the dose and concentration of the administered L arginine-HCl solution and also the strain of mice.

## Introduction

Acute pancreatitis (AP) is one of the most challenging diseases of the pancreas. The main causes of AP are heavy alcohol consumption and gallstone disease [1]. The prevalence of both etiological factors shows increasing tendency making the disease a widespread problem. Although, 80% of cases are mild, the remaining 20% of the patients suffer from severe AP form. The mortality of the latter group can reach 50%.

To study the pathomechanism and therapy of AP, usually animal models such as the secretagouge-induced, the retrograde injection of bile acid-induced, the choline-deficient ethionine-supplemented (CDE) diet-induced and the basic amino acid (L-arginine, L-ornithine or L-lysine)-induced AP models are used [2][3]. None of these models is perfect; each has its own advantages and disadvantages. The most commonly investigated AP model is induced by repetitive injections of secretagouges (like cholecystokinin or caerulein). This treatment causes mild, edematous pancreatitis in rats and severe inflammation and cell damage in mice [which require six to ten intraperitoneal (i.p.) injections given at hourly intervals]. [4] The retrograde injection of bile acids produces patchy necrosis in the pancreatic head, but it involves the use of general anesthesia and a surgical procedure. The CDE diet-induced AP model, which causes severe necrotizing pancreatitis with hemorrhage, works only in young female mice, and this diet is very expensive. The L-arginine-induced AP model was first described by Mizunuma et al. [5] and Tani et al. [6] in rats. A single i.p. injection of 5 g/kg L-arginine selectively destroyed nearly all of the pancreatic acinar cells [5]. It was not until 2007 that Dawra et al. [7] characterized the L-arginine-induced model (2×4 g/kg i.p.) in BALB/c and C57BL/6 mice as well. This basic amino acid-induced pancreatitis model is becoming increasingly popular as it is cheap, non-invasive and easy to induce as it only requires two i.p. injections to produce severe necrotizing disease [8][9][10]. However, carefully looking through the literature, marked differences in disease severity could be observed in mice. In fact, while setting up the L-arginine-induced AP model in BALB/c mice, we found a relatively low rate (around 15%) of pancreatic necrosis in response to the basic amino acid, whereas others have detected much higher rates (up to 55%) in C57BL/6 mice [11][12][13].

Our overall aim was to characterize this increasingly popular AP model so that it can be used with the greatest efficiency. Therefore, we decided to test the effects of L-arginine doses and concentrations in different mouse strains. In addition, the gender dependence of this basic amino acid-induced AP model was examined.

## Materials and Methods

### Ethics

All experiments were conducted in compliance with the *Guide for the Care and Use of Laboratory Animals* (National Academies Press, Eight Edition, 2011), and were approved by the Institutional Animal Care and Use Committee of the University of Szeged (I-74-3/2012 MÁB) and also by an independent committee assembled by national authorities (XII./3773/2012.).

### Animals and materials

Male and female mice weighing 20–25 g were used. FVB/n, C57BL/6 and BALB/c mouse strains were from Charles Rivers Laboratory. The animals were kept at a constant room temperature of 23°C with a 12 hour light–dark cycle and were allowed free access to water and standard laboratory chow for rodents (Biofarm, Zagyvaszántó).

All chemicals were purchased from Sigma-Aldrich (Budapest, Hungary) unless indicated otherwise.

### Induction of acute pancreatitis

L-arginine-HCl was dissolved in physiological saline (PS), and its pH was set to 7.4 with NaOH. The L-arginine solution was prepared freshly before each experiment. Different concentrations (5, 8, 10 and 30%) and doses (2×4, 3×3 and 4×2.5 g/kg, administered at hourly intervals) of L-arginine-HCl were administered i.p. Control animals were treated i.p. with PS instead of L-arginine. Mice were sacrificed at the peak of pancreatic injury, 72 h after the first i.p. injection following anesthesia with i.p. 85 mg/kg pentobarbital (Bimeda MTC, Cambridge, Canada). Animals were exsanguinated through cardiac puncture, the pancreas was rapidly removed and trimmed from fat and lymph nodes. Parts of the pancreatic tissue were immediately frozen in liquid nitrogen then stored at -80°C until biochemical assays were performed. Another part of the pancreas was fixed in 6% neutral formaldehyde solution for histological analysis. Blood samples were centrifuged at 2500 RCF for 15 min at 4°C and the serum was stored at -20°C until use.

The mice became sluggish and lethargic soon after the i.p. L-arginine-HCl injections. Some mice recovered within 12 h after the injections, but others remained unwell. Based on preliminary experiments and literature data, since the core temperature (measured rectally with a digital thermometer, (TFC-305P, ONETEMP) of the latter mice decreased to critical levels (27–29 °C) 12 h after the L-arginine injections, these animals were euthanized by pentobarbital overdose (200 mg/kg i.p.) to minimize suffering [14][15]. The postmortem macroscopic and histological analysis proved that these mice did not suffer from AP (data not shown). The surviving mice gradually recovered from the L-arginine injections and either developed necrotizing AP or remained AP-free by 72 h (when the experiment was terminated). The effectivity rate of AP induction is defined as (the number of mice which showed signs of AP divided by the total number of mice injected with L-arginine) × 100. The signs of AP on histological analysis included pancreatic edema, acinar cell damage, and inflammatory infiltration. The euthanized animals and those that did not show any signs of AP according to the histological analysis were excluded from the measurements (except in BALB/c mice treated with 2×4 g/kg 5% L-arginine-HCl which recovered from the L-arginine-HCl injections and none of the animals demonstrated signs of AP). Due to ethical issues, particularly in case of groups that needed euthanasia in large proportions, the laboratory and histological analyses were performed on a lower number of mice (in order to avoid the unnecessary death of animals).

### Laboratory measurements

Serum amylase activity was measured with a colorimetric kinetic method using a commercial kit purchased from Diagnosticum ZRt. (Budapest, Hungary).

To evaluate the water content of the pancreas, the pancreatic wet weight was measured, then the tissue was dried for 24 h at 100°C and the dry weight was also measured. The dry weight (DW) and wet weight (WW) ratio was calculated as: (1-DW/WW)*100.

Pancreatic myeloperoxidase (MPO) activity is a hallmark of leukocytic infiltration and was measured according to Kuebler et al. [16]. MPO activities were normalized to total protein content measured by the Lowry method [17].

To determine the extent of inflammatory response in the pancreata, we measured interleukin (IL)-1β levels by a commercial ELISA kit from R&D Systems (Minneapolis, MN, USA) as described previously [18].

### Histological examination

Histological samples were prepared and stained with hematoxylin and eosin. Pancreatic sections were analysed and scored by a pathologist blinded to the experimental protocol. Edema was scored from 0–3 points (0: none; 1: patchy interlobular; 2: diffuse interlobular; 3: diffuse interlobular and intraacinar), leukocytic infiltration from 0–3 points (0: none; 1: patchy interlobular; 2: diffuse interlobular; 3: diffuse interlobular and intraacinar), the percentage of acinar cell necrosis was evaluated by ImageJ software (NIH, Bethesda, MD, USA). Normal, non-AP pancreatic samples from L-arginine-treated mice were eventually excluded from the data analysis.

### Statistical analysis

Data are presented as means ± SEM. Differences in euthanasia rates (mortality) were determined by chi square test with Yates correction. Laboratory and histological parameters were evaluated by using the one–way analysis of variance (ANOVA) followed by Bonferroni post hoc test, if the distribution of data was normal. Kruskal-Wallis non-parametric test with Dunnett’s multiple comparison post hoc test was used, if the distribution of data was not normal. P<0.05 was accepted as statistically significant.

## Results

### The effects of L-arginine concentration on the development of acute pancreatitis in BALB/c, C57BL/6 and FVB/n mice

**Fig 1:**
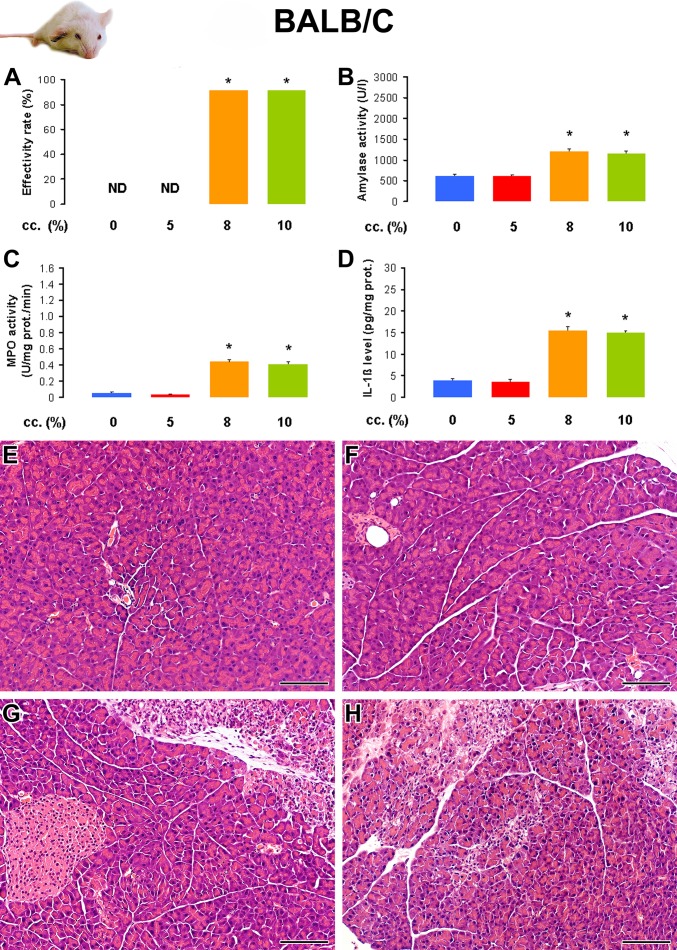
The effect of L-arginine-HCl solution concentration on the induction of acute pancreatitis (AP) in BALB/c mice. Animals were injected i.p. with different concentrations (5–10%) of L-arginine-HCl (pH = 7.4) at a dose of 2×4 g/kg (with an hourly interval) and were sacrificed 72 h after the first injection. The control animals (0% L-arginine) were treated with physiological saline (PS). The number of total / sacrificed non-AP / sacrificed AP mice (based on histological analysis) were: 8 / 8 / 0 in the PS group, 12 / 12 / 0 in the 5% L-arginine-treated group, 12 / 1 / 11 in the 8% L-arginine-treated group and 12 / 1 / 11 in the 10% L-arginine-treated group. Animals that did not show any signs of AP according to histological analysis (sacrificed non-AP mice) were excluded from the measurements (except in mice injected i.p. with 2×4 g/kg 5% L-arginine-HCl solution, which did not demonstrate any signs of AP). **A**. The effectivity rate of AP induction is defined as (the number of mice which showed signs of AP divided by the total number of mice injected with L-arginine) x 100. **B**. Serum amylase activities, **C**. pancreatic myeloperoxidase (MPO) activities, and **D**. pancreatic interleukin (IL)-1β levels were determined from the control group and animals with AP (except in the group treated with 2×4 g/kg 5% L-arginine-HCl). The histological pictures show representative H&E staining of pancreatic samples from mice treated i.p. with **E**. PS, **F**. 5% L-arginine, **G**. 8% L-arginine and **H**. 10% L-arginine. Scale bar = 100 μm.*: p<0.05 vs the control group and 5% L-arginine-treated group.

In the original article by Dawra et al. [7], the 2×4 g/kg L-arginine-HCl dose was administered as an 8% L-arginine solution. Since this involves the injection of a relatively large amount of fluid, first we wanted to characterize the effects of various L-arginine concentrations (5%, 8%, 10% and 30%) on the development of AP in BALB/c mice. According to preliminary experiments, the administration of 30% L-arginine solution (which is commonly used in rats for AP induction) greatly increased the need of euthanasia (over 90% in all mouse strains), so we did not proceed with this concentration any further. Interestingly, whereas treatment of mice with 2×4 g/kg 5% L-arginine solution did not cause any mortality or pancreatic damage (therefore, its effectivity rate was 0) in the BALB/c strain, the effectivity rate of the 8 and 10% L-arginine-treated groups was over 90% (Fig. 1A).

Fig. 1B-H and Table 1 show various laboratory and histological parameters that confirm the development of experimental AP in the 8 and 10% L-arginine-treated groups, whereas no AP was seen in the 0 and 5% treated groups. Notably, the extent of acinar cell damage was relatively mild in pancreatitic animals. There were no significant differences in the measured parameters of the 8 and 10% treated groups, so to decrease the volume load, we used a 10% L-arginine solution for further studies.

**Table 1:** The i.p. administration of 2×4 g/kg L-arginine-HCl (10%) induces acute pancreatitis with a low degree of necrosis in BALB/c mice.

**L-arginine cc. (%)**	(1-D.W./W.W.)*100 (%)	**Leukocytic infiltration (0–3)**	**Necrosis (%)**
0	69.0 ± 0.9	0	0
5	70.3 ± 0.4	0	0
8	76.2 ± 0.7 *	1.4 ± 0.2 *	13 ± 2 *
10	75.3 ± 0.8 *	1.5 ± 0.22 *	16 ± 2 *

Animals were treated as indicated in the legend of Fig. 1. The effects of L-arginine-HCl solution concentration on the induction of AP are shown in BALB/c mice based on measurements of pancreatic water content, leukocytic infiltration score and necrosis rate. L-arginine-treated mice that had no pancreatic inflammation on histology at the time of sacrifice were excluded from the data analysis. *: p<0.05 vs the control (0% L-arginine). Control group: n = 8, 5% group: n = 12, 8% group: n = 11, 10% group: n = 11.

**Fig 2:**
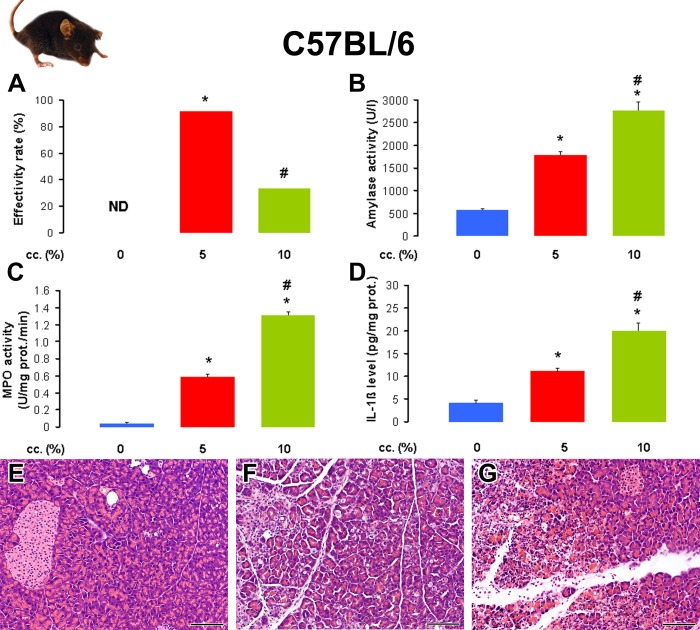
The effect of L-arginine-HCl solution concentration on the induction of acute pancreatitis in C57BL/6 mice. Animals were treated with PS or 2×4 g/kg L-arginine-HCl i.p. as described in the legend of Fig. 1. The number of total / euthanized / sacrificed non-AP / sacrificed AP mice (based on histological analysis) were: 8 / 0 / 8 / 0 in the PS group, 12 / 0 / 1 / 11 in the 5% L-arginine-treated group, 12 / 7 / 1 / 4 in the 10% L-arginine-treated group. The euthanized animals and those that did not show any signs of AP according to histological analysis (sacrificed non-AP mice) were excluded from the measurements. **A**. The effectivity rate of L-arginine-induced AP in C57BL/6 mouse strain. **B**. Serum amylase activities, **C**. pancreatic MPO activities, and **D**. pancreatic IL-1β levels were determined from the control group and animals with AP. The histological pictures show representative H&E staining of pancreatic samples from mice treated i.p. with **E**. PS, **F**. 5% L-arginine, and **G**. 10% L-arginine. Scale bar = 100 μm. p<0.05 vs the *: control group or the #: 5% L-arginine-treated group.

Next, we tested the effects of L-arginine concentration on the C57BL/6 mouse strain. Although the effectivity rate of AP induction was 93% in the 5% L-arginine-treated group, it was only 33% in the 10% L-arginine-treated group (Fig. 2A). The low effectivity rate in the latter group was caused by the high euthanasia rate. Both laboratory and histological parameters confirmed the presence of AP in the groups treated with 5 and 10% L-arginine vs the physiological saline-treated control group (Fig. 2B-H and Table 2). AP was more severe in the 10% vs 5% L-arginine-treated group.

**Table 2:** The i.p. administration of 2×4 g/kg L-arginine-HCl induces a more severe necrotizing acute pancreatitis when a 10% vs 5% L-arginine solution is injected in C57BL/6 mice.

**L-arginine cc. (%)**	(1-D.W./W.W.)*100 (%)	**Leukocytic infiltration (0–3)**	**Necrosis (%)**
0	68.8 ± 0.5	0	0
5	74.6 ± 0.7 *	1.0 ± 0.1 *	16 ± 2 *
10	84.8 ± 2.6 * #	2.8 ± 0.3 * #	54 ± 4 * #

Animals were treated as indicated in the legend of Fig. 2. Pancreatic water content, leukocytic infiltration score and necrosis percentages were determined as indicated in the Methods. Mice that were euthanized 12 h after the injections of L-arginine (not related to AP induction), or L-arginine-treated mice that had no pancreatic inflammation on histology at the time of sacrifice were excluded from the data analysis. p<0.05 vs the *: control (0% L-arginine) or the #: 5% L-arginine-treated group. Control group: n = 8, 5% group: n = 11, 10% group: n = 4. Note that the low n number in the 10% L-arginine-treated group was due to ethical issues (to avoid the unnecessary death of animals).

**Fig 3:**
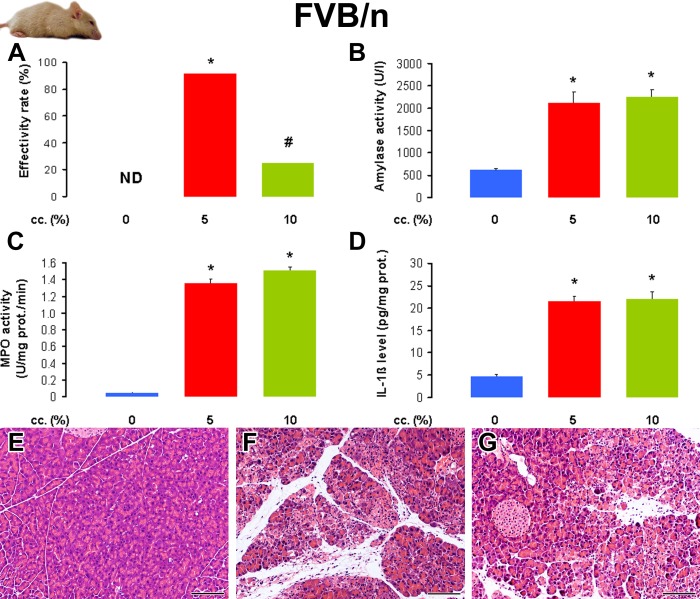
The effect of L-arginine-HCl solution concentration on the induction of acute pancreatitis in FVB/n mice. Animals were treated with PS or 2×4 g/kg L-arginine-HCl i.p. as described in the legend of Fig. 1. The number of total / euthanized / sacrificed non-AP / sacrificed AP mice (based on histological analysis) were: 8 / 0 / 8 / 0 in the PS group, 12 / 0 / 1 / 11 in the 5% L-arginine-treated group, 12 / 8 / 1 / 3 in the 10% L-arginine-treated group. The euthanized animals and those that did not show any signs of AP according to histological analysis (sacrificed non-AP mice) were excluded from the measurements. **A**. The effectivity rate of L-arginine-induced AP in FVB/n mouse strain. **B**. Serum amylase activities, **C**. pancreatic MPO activities, and **D**. pancreatic IL-1β levels were determined from the control group and animals with AP. The histological pictures show representative H&E staining of pancreatic samples from mice treated i.p. with **E**. PS, **F**. 5% L-arginine, and **G**. 10% L-arginine. Scale bar = 100 μm. p<0.05 vs the *: control group or the #: 5% L-arginine-treated group.

Similarly to that observed in C57BL/6 mice, in the FVB/n mouse strain the effectivity rate in the 5% L-arginine-treated groups was 92%; however, it was only 25% in the 10% L-arginine-treated group (Fig. 3A). Laboratory and histological parameters were significantly elevated in the 5 and 10% L-arginine-treated groups, compared to the control group (Fig. 3B-H and Table 3). However, there were no significant differences between the 5 and 10% L-arginine-treated groups. Administration of 5 and 10% L-arginine caused similar degree of pancreatic inflammation.

**Table 3:** The i.p. administration of 2×4 g/kg L-arginine-HCl (10%) induces a similar degree of severe necrotizing acute pancreatitis in FVB/n mice treated with 5% or 10% L-arginine-HCl solution.

**L-arginine cc. (%)**	(1-D.W./W.W.)*100 (%)	**Leukocytic infiltration (0–3)**	**Necrosis (%)**
0	69.3 ± 0.6	0	0
5	77.7 ± 0.8 *	2.0 ± 0.2 *	43 ± 7 *
10	84.2 ± 0.4 * #	2.3 ± 0.3 *	48 ± 6 *

Animals were treated as indicated in the legend of Fig. 3. Pancreatic water content, leukocytic infiltration score and necrosis percentages were determined as indicated in the Methods. Mice that were euthanized 12 h after the injections of L-arginine (not related to AP induction), or L-arginine-treated mice that had no pancreatic inflammation on histology at the time of sacrifice were excluded from the data analysis. p<0.05 vs the *: control (0% L-arginine) or the #: 5% L-arginine-treated group. Control group: n = 8, 5% group: n = 11, 10% group: n = 3. Note that the low n number in the 10% L-arginine-treated group was due to ethical issues (to avoid the unnecessary death of animals).

### Dose-response of L-arginine administration in C57BL/6 and FVB/n mice

**Fig 4:**
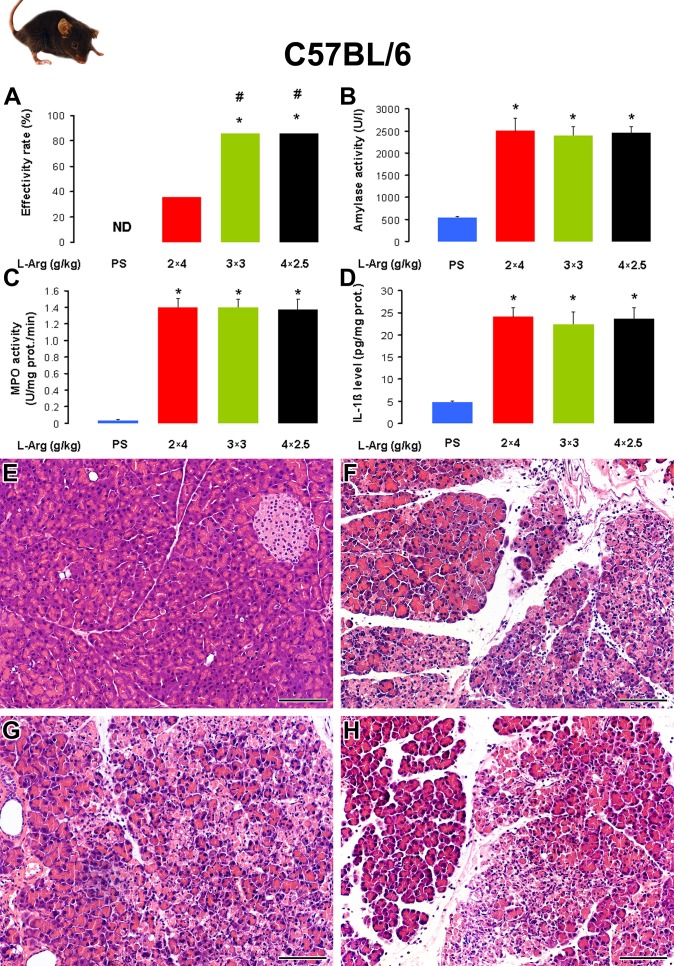
The dose-response of L-arginine-HCl administration in C57BL/6 mice. Mice were treated with PS or different i.p. doses (2×4, 3×3 and 4×2.5 g/kg given at 1-h intervals) of L-arginine-HCl (10%, pH = 7.4) and were sacrificed 72 h after the first injection. The number of all total / euthanized / sacrificed non-AP / sacrificed AP mice (based on histological analysis) were: 10 / 0 / 10 / 0 in the PS group, 14 / 8 / 1 / 5 in the 2×4 g/kg L-arginine-treated group, 14 / 1 / 1 / 12 in the 3×3 g/kg L-arginine-treated group, 14 / 0 / 2 / 12 in the 4×2.5 g/kg L-arginine-treated group. The euthanized animals and those that did not show any signs of AP according to histological analysis (sacrificed non-AP mice) were excluded from the measurements. **A**. The effectivity rate of L-arginine-induced AP C57BL/6 mouse strain. **B**. Serum amylase activities, **C**. pancreatic MPO activities, and **D**. pancreatic IL-1β levels were determined from the control group and animals with AP. Representative H&E staining of pancreatic samples from mice treated i.p. with **E**. PS, **F**. 2×4 g/kg L-arginine-HCl, **G**. 3×3 g/kg L-arginine-HCl and **H**. 4×2.5 g/kg L-arginine-HCl. Scale bar = 100 μm. p<0.05 vs the *: control group or the #: 2×4 g/kg L-arginine-treated group. Note that significance was borderline (p = 0.053) in case of PS- vs 2×4 g/kg L-arginine-treated groups.

BALB/c mice proved to be rather resistant against mortality and AP injury caused by i.p. injection of 2×4 g/kg L-arginine, whereas the other two mouse strains seemed to be much more susceptible. We figured that by using other L-arginine doses, we can reduce adverse effects and increase pancreatic damage in C57BL/6 and FVB/n mice. Fig. 4 and Table 4 show the effectivity rate and laboratory and histological parameters in response to i.p. injection of 2×4, 3×3, or 4×2.5 g/kg L-arginine (10%) in C57BL/6 mice. The 2×4 g/kg L-arginine dose resulted in a low effectivity rate, because of the numerous euthanized mice. In contrast, the effectivity of AP induction in response to administration of 3×3 g/kg or 4×2.5 g/kg L-arginine-HCl was significantly higher vs the 2×4 g/kg L-arginine-HCl treated group (Fig. 4A). The severity of AP was not significantly different in the L-arginine-treated groups according to histological and laboratory parameters. Of note, the i.p. injection of 5×2 g/kg L-arginine-HCl at hourly intervals did not cause any signs of AP (n = 5, data not shown). We found similar dose-response (2×4, 3×3, or 4×2.5 g/kg L-arginine-HCl) results in FVB/n mice (data are not shown). There were no significant differences between the effects of different L-arginine doses on the severity of AP.

**Table 4:** Acute pancreatitis severity in response to the i.p administration of 2×4, 3×3 or 4×2.5 g/kg L-arginine-HCl (10%) is similar in C57BL/6 mice.

**L-arginine dose (g/kg)**	(1-D.W./W.W.)*100 (%)	**Leukocytic infiltration (0–3)**	**Necrosis (%)**
0	67.7 ± 1.1	0	0
2x4	82.8 ± 2.2 *	2.6 ± 0.3 *	60 ± 12 *
3x3	81.4 ± 0.9 *	2.3 ± 0.2 *	52 ± 6 *
4x2.5	80.8 ± 0.8 *	2.3 ± 0.2 *	50 ± 5 *

Mice were treated as indicated in the legend of Fig. 4. Pancreatic water content, leukocytic infiltration score and necrosis percentages were determined as indicated in the Methods. Mice that were euthanized 12 h after the injections with L-arginine, or L-arginine-treated mice that had no pancreatic inflammation on histology at the time of sacrifice were excluded from the data analysis. *: p<0.05 vs the control (0 g/kg L-arginine). Control group: n = 10, 2×4 g/kg L-arginine-treated group: n = 5, 3×3 g/kg L-arginine-treated group: n = 12, 4×2.5 g/kg L-arginine-treated group: n = 12.

### The severity of L-arginine-induced acute pancreatitis is similar in male and female mice

As some AP models show gender dependency (e.g. female mice are more susceptible to CDE diet-induced AP), we wanted to test the effects of L-arginine administration in male and female mice. C57BL/6 mice were subjected to hourly i.p. injections of 3×3 g/kg 10% L-arginine-HCl. The effectivity rate of AP induction was 87.5% in male (n = 8) and 100% in female (n = 8) mice, which was not significantly different. The laboratory and histological parameters were similar to that shown in Fig. 2B-G.

## Discussion

An important finding of our experiments is it that different mouse strains have varying sensitivities (resulting in AP, no pancreatic damage, or death) to administration of L-arginine-HCl (pH = 7.4) (Table 5.). In FVB/n and C57BL/6 mice, which are commonly used strains for generating transgenic animals, the originally published i.p. 2×4 g/kg L-arginine-HCl dose may not be appropriate and the use of other doses (like 3×3 g/kg or 4×2.5 g/kg) may be better for the induction of AP. Furthermore, the concentration of the administered L-arginine-HCl solution makes a huge difference in whether the animals survive the treatment. High L-arginine concentrations like 30%, which are well tolerated by rats actually kill mice, thus lower (5–10%) concentrations should be injected even if this means considerably more fluid volume. Interestingly, we could not detect any differences in AP severity of male and female mice.

**Table 5:** Summary of the effectivity rates and the severity of acute pancreatitis in different mouse strains.

**Strain**	**FVB/n**			**C57Bl/6**			**Balb/c**		
Dose (g/kg)	2×4	2×4	3×3 or 4×2.5	2×4	2×4	3×3 or 4 ×2.5	2×4	2×4	3×3 or 4 ×2.5
Cc (%)	5	10	10	5	10	10	5	8 or 10	10
Effectivity rate	+++	+	++	+++	+	+	0	+++	++
Severity of AP	+++	+++	++	+	+++	+++	0	+	+

Mouse strains, L-arginine doses and concentrations are shown.

Bold = not suggested, Italics = data not shown in the article.

Considering the adverse effects of large L-arginine doses (that would have lead to the death of animals without euthanasia), the sensitivity of FVB/n and C57BL/6 strains to the basic amino acid was much higher than that of BALB/c mice. In fact, it seems that the most L-arginine resistant mouse strain is the BALB/c. In this strain, we found no mortality in response to i.p. injection of 2×4 g/kg L-arginine-HCl. In accord with our observations, Dawra et al. [7] (who have also used BALB/c mice) reported no mortality in their original study. In contrast, we had to utilize euthanasia in a large proportion of FVB/n and C57BL/6 mice after the i.p. administration of 2×4 g/kg L-arginine-HCl. The cause of death is unrelated to AP and may be due to metabolic [like severe metabolic acidosis detected in rats by Bohus et al. [19]] or central nervous system effects. The reasons for the above mentioned discrepancies are not clear, but the dose response of rats to i.p. administration of L-arginine [19] and L-lysine [20] can be variable even in one animal strain, although no such effects have been reported in mice. Sprague-Dawley rats treated with a high dose (4 g/kg) of i.p. L-arginine caused weak, normal and strong response [19]. Weak responders had no marked alterations vs the saline-treated control group, whereas strong responders died. In our hands, the administration of 3.5 g/kg L-arginine causes marked pancreatic inflammation and cell damage, but no mortality [18]; however, we have also observed a small percentage of mortality with the 4 g/kg L-arginine-HCl dose in male Wistar rats (unpublished data). Similarly to the data of Bohus et al.[19], the i.p. injection of large L-lysine doses caused nil to severe pancreatic damage or death of rats [20]. Based on the above mentioned data, we speculate that there may be differences in basic amino acid sensitivities even within the same mouse strain.

Most commonly, an i.p. dose of 2×4 g/kg L-arginine-HCl is used for the induction of AP, which was initially used in the current study as well. The utilization of higher [21][22], and some rather odd [23][24][25][26][27] L-arginine doses have also been reported. Previously published L-arginine doses ranging from 1×0.000004 g/kg [25] to 2×2.25 g/kg [27] administered in mice do not cause any signs of AP in our hands (data not shown). Similarly, Dawra et al. [7] could not detect any significant changes in serum amylase activity, and pancreatic MPO activity and histopathology in response to i.p. 2×2 g/kg or 2×3 g/kg L-arginine-HCl in BALB/c mice. However, we have clearly shown that the administration of 3×3 g/kg or 4×2.5 g/kg L-arginine-HCl induced severe AP in the majority of FVB/n and C57BL/6 mice, but caused a marked reduction of adverse effects (resulting in euthanasia) vs the 2×4 g/kg dose. When we administered the same L-arginine doses in the three mouse strains, AP severity was nearly the same in FVB/n and C57BL/6 mice, but it was markedly less in BALB/c mice. The strain-dependent susceptibility to cerulein- and CDE diet-induced AP was reported by Wang et al. [4]. The severity of experimental pancreatitis in their study was moderate in BALB/c and mild in C57BL/6J mice. This difference in susceptibility was attributed to a positive relationship with proteinase, serine, 1 (*PRSS1*) expression and a negative relationship with serine protease inhibitor, Kazal type 3 (*SPINK3*) expression. In L-arginine-induced AP, this may not be the case since disease severity was significantly higher in C57BL/6 vs BALB/c mice.

The effects of the estrous cycle on AP development have always been a concern of researchers, thus to exclude such potential effects, usually male animals are used. In fact, it has long been known that in case of the CDE diet model, the effects are gender specific, female mice are much more sensitive to treatment [28]. Therefore, we also tested the potential gender-specific effects of L-arginine treatment, but we found no significant differences in AP severity of male vs female C57BL/6 mice.

Taken together, it is evident that setting up the L-arginine-induced AP model in mice is quite challenging. Overall, it seems that the borderline between the effective (AP inducing) and lethal doses of L-arginine is much thinner in mice vs rats. There are several important factors that need to be considered such as the concentration and dose of the injected L-arginine-HCl solution and also the strain of mice. The proper dosing of L-arginine to induce AP should be tested by each laboratory and in each mouse strain. The reasons for the differences in L-arginine sensitivity of mice remain to be investigated.
